# Alpine Grassland Ecological Restoration Approaches Shape Insect Trophic Guild Diversity: A Multi-Dimensional Assessment from Alpha to Dark Diversity

**DOI:** 10.3390/insects16111140

**Published:** 2025-11-07

**Authors:** Kuanyan Tang, Hongru Yue, Haijuan Qu, Yifang Xing, Bingshuang Qin, Aosheng Wang, Kejian Lin, Kun Shi, Ning Wang

**Affiliations:** 1 Institute of Grassland Research, Chinese Academy of Agricultural Sciences, Hohhot 010010, China; tangky5920@163.com (K.T.); 17393154662@163.com (H.Y.); 18647720822@163.com (H.Q.); gezelligbeau@yeah.net (Y.X.); 19999726980@163.com (B.Q.); wanggaoder@163.com (A.W.); linkejian@caas.cn (K.L.); 2 Wildlife Institute, School of Ecology and Nature Conservation, Beijing Forestry University, Beijing 100083, China

**Keywords:** alpine grassland, ecological restoration, insect functional groups, zeta diversity, dark diversity

## Abstract

**Simple Summary:**

The degradation of high-altitude grasslands on the Tibetan Plateau threatens both the environment and local livelihoods. Insects play essential roles in keeping these ecosystems healthy, but it was unclear how different grassland restoration methods affect insect communities. Our study compared four approaches: grazing exclusion fencing, no-till reseeding, planting grass, and traditional grazing. We found that no-till reseeding supported the most diverse and balanced insect community, benefiting predators, plant-eaters, and decomposers alike. Grazing exclusion fencing helped plant-eating insects but was less effective for other groups, while planting grass even reduced the diversity of some important insects. Our results suggest that no-till reseeding is the most effective method to restore both insect diversity and overall grassland health. This approach can help the recovery of fragile ecosystems while supporting sustainable grazing practices.

**Abstract:**

The severe degradation of alpine grasslands on the Qinghai–Tibet Plateau poses a significant threat to regional ecological security. While insects are critical for ecosystem functions, their responses to restoration measures in these fragile habitats are poorly documented. This study assessed the initial impacts of four restoration approaches—grazing exclusion fencing (FE), no-till reseeding (FR), planting grass (GC), and grazing control (CK)—on insect trophic guilds (herbivores, predators, saprophagous, and omnivores) in the Qilian Mountains. Using a multi-dimensional indicator (alpha, zeta, and dark diversity), we systematically assessed community assembly and recovery potential. The results revealed the following: (1) FE supported the highest insect abundance, dominated by phytophagous insects. FR significantly enhanced species’ richness and diversity across multiple functional groups (*p* < 0.05). GC significantly increased the richness of omnivorous insects, but caused a significant decrease in the Shannon–Wiener index for saprophagous insects (*p* < 0.05). (2) Zeta diversity revealed stable, widespread-species-dominated communities under FR and FE, while CK and GC favored rare-species-driven succession. Dark diversity analysis indicated high recovery potential for phytophagous insects under FR and FE, while GC enhanced saprophagous latent diversity. However, we emphasize that mechanistic interpretations require further validation. Our findings highlight no-till reseeding as a promising initial strategy, though longer-term studies are essential to evaluate successional trajectories and establish definitive management protocols for alpine grassland restoration.

## 1. Introduction

Grasslands, covering approximately 40% of the Earth’s terrestrial surface, play an indispensable role in global carbon cycling, hydrological regulation, and biodiversity maintenance [1,2,3]. These ecosystems not only provide critical ecological services but also support the sustainable development of global livestock production [4]. However, they are facing unprecedented degradation due to climate change and human activities, especially in fragile alpine regions like the Qinghai–Tibet Plateau [5]. The United Nations’ “Decade on Ecosystem Restoration 2021–2030” initiative underscores the urgency and importance of advancing grassland restoration research [6].

The Qinghai–Tibet Plateau, often referred to as the “Roof of the World” harbors unique alpine grassland ecosystems with exceptional ecological functions [7]. Recent studies have shown that the region is the source of major Asian rivers and stores a significant quantity of soil organic carbon [8]. However, climate warming is accelerating the degradation of over 90% of alpine grasslands, which poses a serious threat to ecosystem function and biodiversity [9]. Despite extensive research on vegetation recovery in grassland restoration, the response of faunal components—particularly insect diversity—remains underexplored in these fragile ecosystems. This knowledge gap is critical given insects’ essential roles in maintaining ecosystem stability through primary productivity, food web dynamics, and nutrient cycling. Restoration measures such as grazing exclusion, no-till reseeding, and artificial grassland establishment are likely to significantly impact insect communities, making them valuable indicators of overall ecosystem health and recovery.

In restoration practices, targeted anthropogenic interventions can significantly accelerate the recovery of degraded ecosystems by enhancing their inherent resilience [10]. Established grassland restoration techniques include grazing exclusion, no-till reseeding, and artificial grassland establishment [11], which primarily address local degradation drivers rather than directly countering climate change effects. However, by reinforcing vegetation structure and supporting functionally diverse insect communities, these measures may improve ecosystem capacity to withstand climate-associated disturbances. Insects serve as sensitive bioindicators of ecosystem condition, with different trophic guilds delivering essential ecological services [12]: herbivores influence plant community composition and primary productivity, predators stabilize food web dynamics [13], and detritivores facilitate nutrient cycling and energy transfer [14,15]. The integrated functioning of these guilds collectively underpins grassland ecosystem stability and adaptive capacity.

Accurately assessing community assembly processes is a major challenge in contemporary biodiversity research. While traditional alpha and beta diversity metrics effectively capture local species richness and compositional variation, they offer limited resolution for discerning the distinct contributions of rare versus widespread species to community organization [16,17]. To address these limitations, we implement an integrated analytical framework combining zeta and dark diversities. Zeta diversity quantifies multi-site species turnover across spatial scales, explicitly revealing how rare and common species drive community transition patterns [18]. Complementarily, dark diversity identifies ecologically suitable but locally absent species, enabling quantification of community completeness and restoration potential [19,20]. Together, these approaches provide mechanistic insights into assembly processes and recovery trajectories that extend beyond conventional metrics, thereby establishing a robust foundation for evaluating restoration efficacy and biodiversity conservation in alpine grasslands and other vulnerable ecosystems.

This study investigates alpine grasslands in the Qilian Mountains of the Qinghai–Tibet Plateau, systematically comparing four restoration approaches representing a gradient from passive recovery (grazing control) to active interventions (grazing exclusion fencing, no-till reseeding, artificial grassland establishment) to examine insect functional group responses. This study aims to (1) evaluate the impact of four restoration measures on insect diversity, (2) assess the relative contributions of rare and widespread species to community aggregation using zeta and dark diversity indicators, and (3) develop evidence-based management strategies for alpine grassland restoration. We hypothesize that active interventions, particularly no-till reseeding, optimize multi-trophic diversity through enhanced resource heterogeneity and habitat complexity. The established multidimensional assessment framework provides novel insights into grassland ecosystem recovery mechanisms while offering valuable implications for global mountain ecosystem conservation.

## 2. Materials and Methods

### 2.1. Study Site and Experimental Design

**Figure 1:**
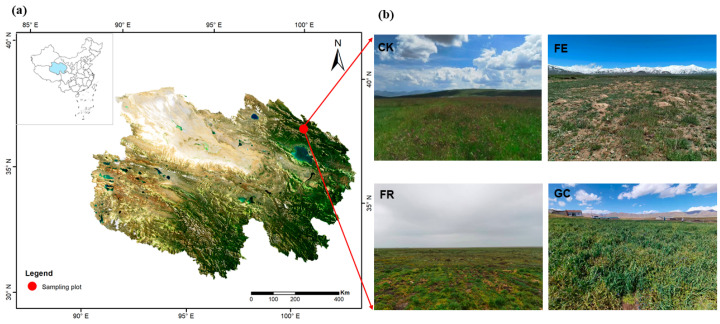
Geographical location of the study site in Qilian County, Qinghai–Tibet Plateau (**a**). Experimental design showing four grassland restoration measures: no-till reseeding (FR), grazing exclusion fencing (FE), planting grass (GC), and grazing management as control (CK) (**b**).

This study was conducted in Mole Town, Qilian County (37°55′ N, 100°13′ E; 3550 m a.s.l.), situated within the northeastern Qinghai–Tibet Plateau (Figure 1a). The area represents a typical alpine meadow ecosystem characterized by a highland continental climate, with mean annual temperature averaging −1.7 °C and annual precipitation ranging from 400~620 mm, predominantly occurring during summer months [21]. The vegetation is dominated by graminoid species, including *Kobresia humilis*, *Elymus nutans*, and *Poa pratensis*, with associated species such as *Stipa purpurea* and *Kobresia pygmaea* [22].

To address severe grassland degradation, we established a controlled experiment in 2020 comparing four restoration strategies along a gradient of intervention intensity. The treatments included (1) no-till reseeding (FR) using broadcast seeding of *Elymus nutans* without soil disturbance, (2) grazing exclusion fencing (FE) allowing natural recovery of *Elymus nutans* and *Poa pratensis*, (3) planting grass (GC) with *Avena sativa* sown in rows, and (4) grazing management (CK) as control, maintaining traditional (unmanaged) stocking density of 5 sheep/hectare. Twelve randomly positioned plots (50 m × 50 m, 0.25 ha) with three replicates per treatment were established (Figure 1b and Figure S1). All treatments were carried out between April and June 2020, and insect sampling was conducted in August 2024. Vegetation development showed distinct treatment-specific patterns: grazing exclusion exhibited partial natural regeneration, reseeded areas maintained mixed perennial grasses, planted areas were dominated by *Avena sativa* monoculture, while grazed controls displayed characteristic degradation signatures typical of regional grazing practices.

### 2.2. Insect Sampling

Insect communities were surveyed during consistent weather conditions (from 15 to 20 August 2024, 09:00–15:00, sunny and windless periods) using standardized sweep nets (40 cm diameter, 110 cm depth) method following established methodologies [23,24,25]. Within each plot center, four transects were established along cardinal directions (SE, NE, SW, NW), 100 sweeps per transect. Critically, the four transects per plot were maintained as separate analytical units throughout our study, preserving within-plot spatial variation rather than pooling samples. This design allowed for assessment of microhabitat heterogeneity within treatments while maintaining the plot as the experimental unit for treatment-level comparisons. All collected specimens were preserved in 95% ethanol centrifuge tubes, brought back to the laboratory, and identified as morphological species through integrated morphological analysis using stereomicroscopes, supported by taxonomic literature, professional books, and specialist consultation [26,27,28].

### 2.3. Data Analysis and Statistics

#### 2.3.1. Alpha Diversity

This study employed a multidimensional analytical framework to systematically evaluate the effects of different restoration approaches on insect community structure and function. The insect assemblage was first classified into four distinct trophic guilds—phytophagous (Ph), predatory (Pr), saprophagous (Sa), and omnivorous (Om)—based on feeding ecology [29]. For each guild, we calculated a suite of complementary alpha diversity indices using the *vegan* R package (version 4.5.1) [23] to capture different aspects of community structure: Margalef richness index (MR) for species richness, Shannon–Wiener index (H’) for overall diversity, Pielou evenness index (J) for distribution evenness, and Simpson index (D) for dominance patterns, following established methodologies [30].

(1) MR=(S−1)/lnN

(2) $$H^{\prime }=-\sum_{i=1}^{S}P_{i}lnP_{i}$$

(3) $$J=H^{\prime }/lnS$$

(4) $$D=1-\sum_{i=1}^{S}P_{i}^{2}$$

where S represents the total number of species in the community, P_i_ represents the proportion of individuals belonging to the i-th species, and N represents the total number of individuals in the community.

#### 2.3.2. Zeta Diversity

Zeta diversity analysis effectively parses the differential contributions of rare and widespread species to community heterogeneity by quantifying multi-site species compositional turnover [18,31]. This method is based on species occurrence rate and the principle of multi-order comparison, where the zeta order (ζ) represents the number of sites being compared: ζ_1_ reflects the average species richness per site, ζ_2_ measures the average number of species shared between any two sites, and ζ_3_ reflects the average number of species shared among any three sites [32]. The construction of zeta decline curves across different orders enables systematic evaluation of how rare versus widespread species differentially contribute to community beta diversity. A steep decline in zeta values with increasing order indicates community turnover driven primarily by rare species, while a gradual decline suggests dominance by widespread species that maintain community connectivity [31].

Furthermore, zeta ratios (ζ_i+1_/ζ_i_) quantify how species co-occurrence probabilities change across spatial scale: ratios approaching unity indicate dominance by widespread species and high spatial coherence in community structure, whereas significantly decreasing ratios with increasing order suggest rare species-driven community turnover [33]. Our analysis was implemented using the *Zetadiv* package in R 4.5.1, where zeta values were computed via the *Zeta.decline.ex()* function with statistical significance assessed through permutation tests.

This methodology rests on two key ecological assumptions: (1) that species distribution patterns across multiple sites contain crucial information about community assembly mechanisms and (2) that zeta decline patterns across different orders can effectively distinguish the relative importance of deterministic versus stochastic processes in community assembly. Through this multi-scale analytical framework, we gain deeper insights into how restoration measures regulate insect spatial distribution patterns by modifying environmental filtering and dispersal limitation.

#### 2.3.3. Dark Diversity

Dark diversity refers to species that are not present in an ecosystem but which belong to its species pool. It can be used to evaluate the potential size of a species pool [19,34]. This study used the Beals index, which is based on species coexistence, to quantitatively assess dark diversity. This method calculates the probability of species presence (P_ki_) within a 50 m × 50 m plot. The formula takes into account species coexistence relationships (M_ij_) and occurrence frequency (n_j_) [35]. To ensure the reliability of the results, a dual verification mechanism was established: first, a threshold was set based on the minimum Beals index value in the presence plots (1%), with species in the absence plots exceeding this threshold being included in hidden diversity [36]. Second, outliers were eliminated through binary transformation. Finally, the potential distribution probability (PDD = ND/NP) was calculated to quantify the potential distribution characteristics of each species [35]. Here, ND represents the occurrence frequency of a species in the dark diversity, and NP represents its occurrence frequency in the species pool. The above calculations were implemented using the Beals function from the *vegan* package.

In order to assess the recovery status of communities undergoing different restoration methods, this study introduced the Community Completeness Index (CCI). This index quantifies a community’s ecological completeness by comparing its actual species richness (SR) with the potential species richness (PR) predicted by latent diversity [37]. The formula is CCI = SR/(SR + PR), with values ranging from 0 to 1 that directly reflect the degree of alignment between community structure and the potential species pool [34]. This provides a standardized metric for evaluating the ecological benefits of different restoration measures. Unlike traditional diversity indices, this method is not sensitive to differences in regional species pools and is therefore particularly suitable for longitudinal comparative studies in restoration ecology.

Treatment effects were analyzed using one-way ANOVA to examine restoration impacts on (1) overall and guild-specific species richness, (2) α-diversity indices, (3) zeta diversity components, and (4) dark diversity probabilities. Before conducting ANOVA, the dataset was tested for normality using the Shapiro–Wilk test and for homogeneity of variance using Levene’s test. When these assumptions were violated, the non-parametric Kruskal–Wallis test was applied as an alternative. Pairwise comparisons were performed using Tukey’s HSD test for ANOVA and Dunn’s test for Kruskal–Wallis analyses, ensuring robust evaluation of differences among treatments [38,39]. This multidimensional analytical approach provided a comprehensive assessment of restoration effectiveness in alpine meadow ecosystems. All statistical analyses were conducted in R (version 4.5.1) using the *agricolae* package [40] for hypothesis testing and the *ggplot2* [41] package for visualizing complex community patterns and restoration outcomes.

## 3. Results

### 3.1. Insect Community Composition

**Figure 2:**
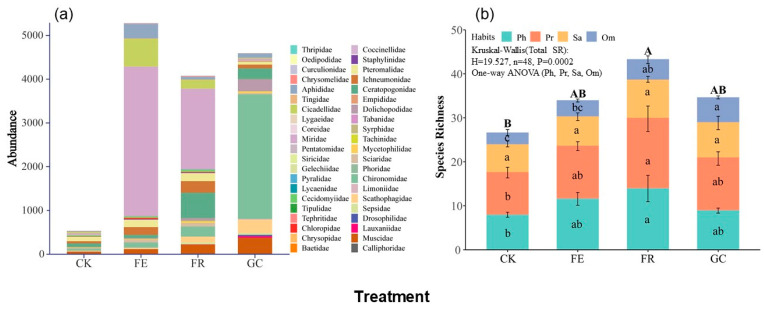
Effects of different grassland restoration measures on insect community composition (**a**) and species richness of functional groups ((**b**), mean values ± SE). Note: grassland restoration measures: no-till reseeding (FR), grazing exclusion fencing (FE), planting grass (GC), and grazing management as control (CK); functional group: phytophagous (Ph), predatory (Pr), saprophagous (Sa), and omnivorous (Om). Different capital letters indicate significant differences among treatments at *p* < 0.05 (Kruskal–Wallis); Different lowercase letters indicate significant differences among treatments, *p* < 0.05 (one-way ANOVA).

We collected a total of 14,487 insect specimens belonging to 88 species, 42 families, and 9 orders during the study. Significant differences in community structure were observed among restoration treatments (*p* = 0.0002) (Figure 2). Grazing exclusion supported the highest overall insect abundance (5280 individuals, 36.45%), with a clear domination by Miridae, Cicadellidae, and Aphididae; planting grass ranked the second highest abundance (4593 individuals, 31.70%), characterized by dipterans, particularly Chironomidae. No-till reseeding (4080 individuals, 28.16%) sustained an intermediate abundance level with balanced representation of Miridae, Ceratopogonidae, and Ichneumonidae. In contrast, grazing management resulted in substantially reduced abundance (534 individuals, 3.69%) and distinct dominance patterns featuring Pteromalidae, Ceratopogonidae, and Ichneumonidae. Detailed numerical data are provided in Supplementary Table S1.

Functional group analysis revealed that no-till reseeding significantly enhanced overall species richness (*p* = 0.0001), particularly for phytophagous (e.g., Miridae) and predatory (e.g., Ichneumonidae, Ceratopogonidae) groups (Figure 2b, Table S2). Planting grass notably increased omnivorous insect species richness (e.g., Muscidae; *p* = 0.008), whereas grazing management reduced species richness in phytophagous and predatory groups (*p* = 0.039 and 0.035). No treatment significantly affected saprophagous insect species richness (*p* = 0.272).

### 3.2. Effects of Restoration Treatments on Insect Alpha Diversity

**Figure 3:**
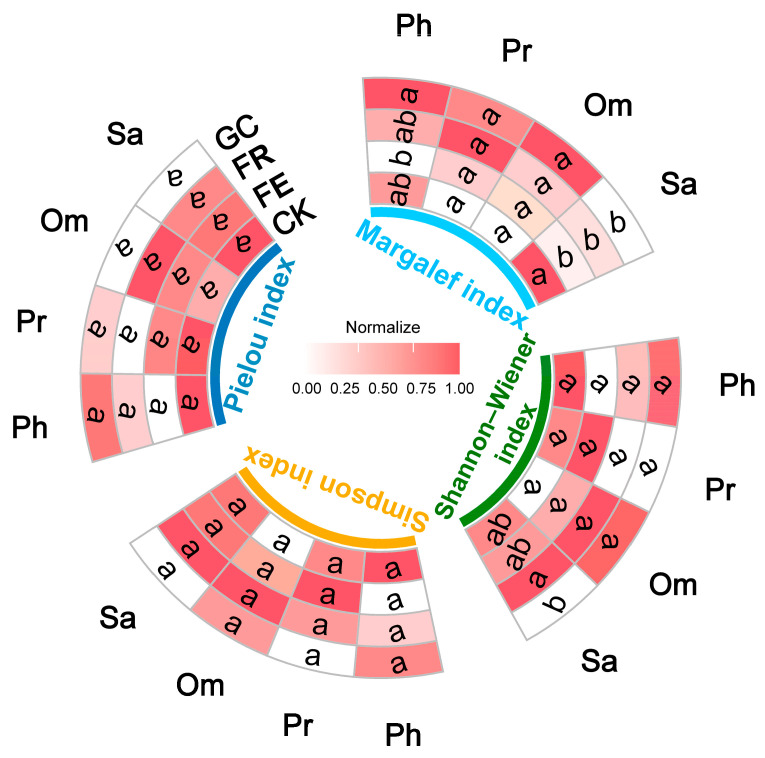
Effects of different grassland restoration measures on insect community species diversity. Note: grassland restoration measures: no-till reseeding (FR), grazing exclusion fencing (FE), planting grass (GC), and grazing management as control (CK); functional group: phytophagous (Ph), predatory (Pr), saprophagous (Sa), and omnivorous (Om). Different lowercase letters indicate significant differences among treatments, *p* < 0.05 (one-way ANOVA). Normalization represents the relative value of the normalized diversity index, used for the relative changes between different treatments.

Alpha diversity indices analysis revealed no significant effects of restoration measures on either Margalef or Shannon–Wiener index for predatory and omnivorous insects (Pr: *p* = 0.9044 and 0.8385; Om: *p* = 0.5741 and 0.6768) (Figure 3, Table S2). For phytophagous insects, planting grass significantly increased the Margalef index (*p* = 0.045) while grazing exclusion fencing reduced it (*p* = 0.036), with no differences in Shannon–Wiener index across treatments. Notably, restoration measures significantly affected saprophagous insect diversity (*p* = 0.0376 and 0.0235, Table S2): grazing management enhanced the Margalef index (*p* = 0.010), no-till reseeding improved Shannon–Wiener index (*p* = 0.049), whereas planting grass decreased the latter (*p* = 0.011). No treatment significantly influenced Simpson or Pielou index across trophic guilds (Ph: *p* = 0.1830 and 0.3820; Pr: *p* = 0.5133 and 0.9243; Sa: *p* = 0.3117 and 0.1665; Om: *p* = 0.9528 and 0.7659, Table S2). The above results indicated that ecological restoration approaches mainly regulate insect community composition and structure by altering species richness (Margalef index) and Shannon–Wiener index.

### 3.3. Effects of Restoration Treatments on Insect Zeta Diversity

**Figure 4:**
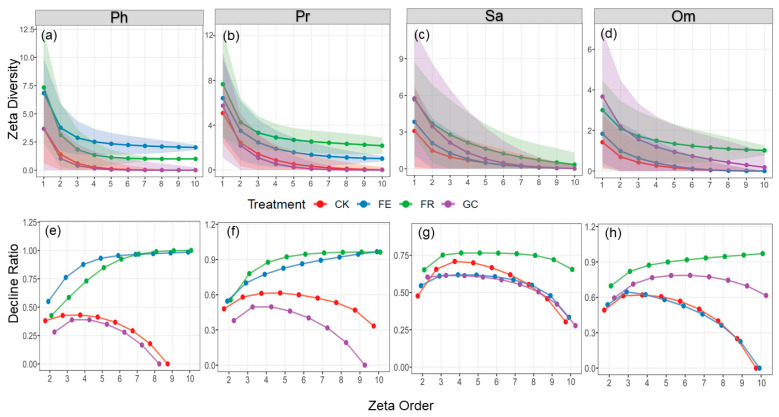
Zeta diversity (**a**–**d**) and zeta ratio (**e**–**h**) plots under different restoration measures for the four functional groups. Note: grassland restoration measures: no-till reseeding (FR), grazing exclusion fencing (FE), planting grass (GC), and grazing management as control (CK); functional group: phytophagous (Ph), predatory (Pr), saprophagous (Sa), and omnivorous (Om). The shaded areas represent 95% confidence intervals.

Our analysis of different treatments for species succession indicated that the zeta diversity of all trophic guilds shows a significant downward trend (Figure 4). There is a sharp decline in the number of species from the 1st to 3rd trophic levels (Figure 4a–d). Specifically, the decline rate of phytophagous insect species under enclosure treatment is relatively slow, while predatory, saprophagous, and omnivorous insects under no-till reseeding treatment exhibit a similar gradual decline pattern. Zeta ratio analysis further revealed that (Figure 4e–h) in phytophagous and predatory insects, the ratios in the fenced and no-till reseeding treatments approached one, indicating stable communities dominated by widespread species; whereas the grazing and planting grass treatments showed an initial increase followed by a decline, reflecting a succession pattern dominated by rare species. Saprophagous insects exhibited a first-increase-then-decrease pattern across all treatments, but the decline was most gradual in the no-till reseeding treatment. Notably, the ratios of phytophagous and omnivorous insects in the grazing treatment, phytophagous and predatory insects in the paddock grassing treatment, and omnivorous insects in the fencing treatment ultimately dropped to zero, indicating complete species succession between treatments. Overall, except for the predatory and herbivorous insects in the fenced and no-till reseeding treatments, all treatments exhibited rapid species turnover and low community similarity.

### 3.4. Effects of Restoration Treatments on Insect Dark Diversity

**Figure 5:**
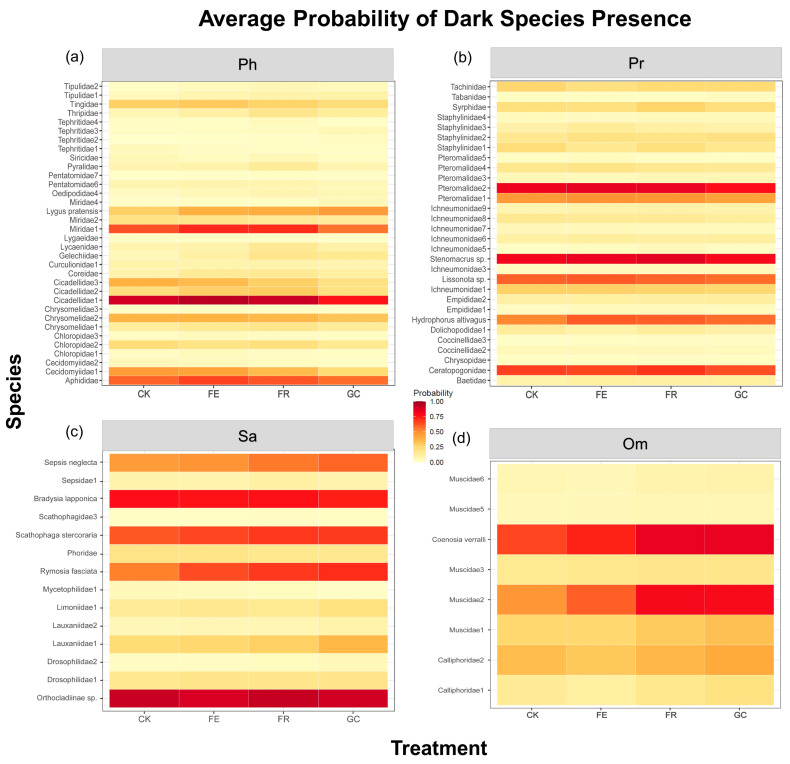
The dark diversity probability of insect functional group species under different grassland restoration measures. Note: grassland restoration measures: no-till reseeding (FR), grazing exclusion fencing (FE), planting grass (GC), and grazing management as control (CK); functional group: phytophagous (Ph), predatory (Pr), saprophagous (Sa), and omnivorous (Om).

Dark diversity probability analysis revealed differential responses of functional groups to ecological restoration measures (Figure 5, Table S3). Herbivorous insect groups with high dark diversity probability (e.g., Cicadellidae and Miridae) exhibited significantly higher values under the grazing exclusion fencing (0.94 and 0.71) and no-till reseeding (0.91 and 0.71), compared to planting grass (0.75 and 0.56, respectively) (Figure 5a). Among predatory insects, parasitoid wasps (Pteromalidae, *Stenomacrus* sp., etc.) showed high sensitivity to no-till reseeding (0.84 and 0.85) (Figure 5b). Saprophagous groups (e.g., Orthocladiinae, *Bradysia lapponica*, and *Scathophaga stercoraria*) exhibited the highest dark diversity probability under planting grass treatment (0.90, 0.74), which was significantly higher than under other treatments (Figure 5c). Omnivorous insects displayed intraspecific differentiation, with sensitive species (e.g., *Coenosia verralli*) exhibiting the highest dark diversity under plating grassing and no-till reseeding treatments (0.84 and 0.84, respectively) (Figure 5d). Overall, predatory and phytophagous insects exhibited fewer species with high dark diversity probabilities than saprophagous and omnivorous insects.

**Figure 6:**
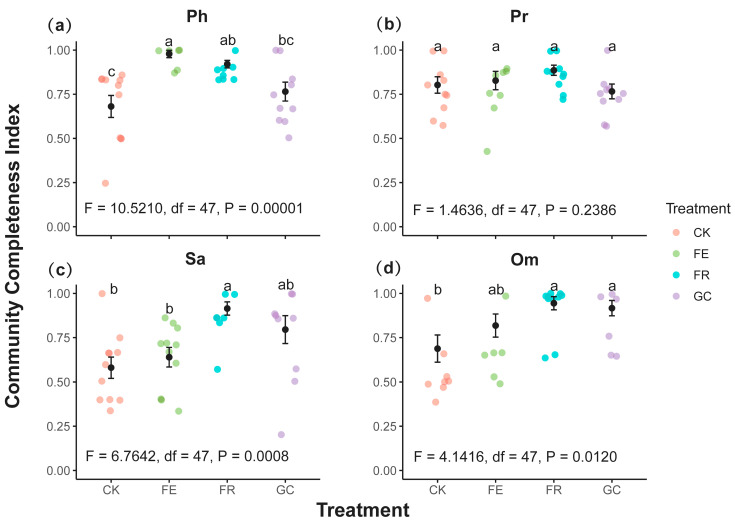
Insect functional community completeness index (CCI) under different grassland restoration measures (mean values ± SE). Note: grassland restoration measures: no-till reseeding (FR), grazing exclusion fencing (FE), planting grass (GC), and grazing management as control (CK); functional group: phytophagous (Ph), predatory (Pr), saprophagous (Sa), and omnivorous (Om). Different lowercase letters indicate significant differences among treatments, *p* < 0.05 (one-way ANOVA).

Community integrity index (CCI) analysis revealed significant differences in the effects of ecological restoration measures on various functional groups (Figure 6). The CCI for phytophagous insects reached its peak in the grazing exclusion fencing (0.98, *p* = 0.00001), significantly higher than in the no-till reseeding (0.92) and the grazing control (0.77) (Figure 6a). Predatory insects performed best under the no-till reseeding (0.89), followed by grazing exclusion fencing (0.83), grazing control (0.78), and planting grassing (0.74) (Figure 6b). Notably, the highest CCI values for both saprophagous and omnivorous insects were observed under no-till reseeding (0.91 and 0.94, respectively, *p* = 0.001 and 0.002), whereas the grazing treatment yielded significantly lower values (*p* = 0.001 and 0.014) (Figure 6c,d). These results indicate that grazing exclusion fencing maintains the community integrity of phytophagous insects, while no-till reseeding enhances the community integrity of all functional groups (predatory, saprophagous, and omnivorous insects).

## 4. Discussion

### 4.1. Differential Effects of Restoration Measures on Insect Functional Group Diversity

Our research findings indicated that grazing exclusion fencing (FE) and no-till reseeding (FR) significantly enhanced insect community species richness and abundance (Figure 2). While grass planting (GC) in enclosures maintained high individual numbers, it exhibited a single dominant group (Chironomidae accounting for 62.03%), potentially due to habitat homogenization resulting from monoculture *Avena sativa* cultivation, which is consistent with previous studies [42,43]. Insect diversity in the grazing management (CK) was the lowest and consistent with the findings of Wang et al. [44], indicating that overgrazing significantly suppresses the recovery potential of insect communities through trampling, vegetation damage, and microhabitat homogenization. No-till reseeding (FR) had the most significant promotional effect on phytophagous and predatory insects, which is consistent with the findings of Cao et al. [42]. These findings demonstrated that no-till reseeding preserves soil structure and increases plant diversity, for example, through the mixed planting of *Elymus nutans* and *Poa pratensis* [45,46]. This provides diverse ecological niches for insects at different trophic levels. Conversely, grazing (CK) significantly reduced the species richness of phytophagous, predatory, and omnivorous insects, but increased the diversity of saprophagous insects (Figure 2b). This phenomenon primarily stems from livestock’s selective grazing behavior, reducing the availability of key host plants and disrupting surface microhabitat structures through trampling activities [47]. The accumulation of manure and urine provides a rich resource base for saprophagous groups, thereby leading to trophic cascading effects and alterations in food web structure [48].

The significant increase in herbivorous insect species richness under planting grass (GC), as indicated by elevated Margalef index values, demonstrates a clear positive response to the concentrated high-quality food resources provided by monoculture oat cultivation (Figure 3). This specialized resource availability created optimal conditions for grass-specialized herbivores, effectively enhancing local diversity within this trophic guild [49]. Conversely, the decreased Margalef index value under grazing exclusion likely resulted from long-term, enclosure-induced succession of the plant community, which led to the dominance of certain grass species (e.g., *Poa pratensis*) and reduced host plant diversity. This, in turn, reduced the abundance of specialist species—a pattern consistent with the resource specialization effect reported by Zhang et al. [50,51]. For saprophagous insects, grazing (CK) significantly increased the Margalef index, primarily due to the rich resource base provided by livestock dung (e.g., attracting Scathophagidae). This finding corroborates the conclusion of Liu et al. [52,53] that grazing disturbance promotes saprophagous diversity. No-till reseeding (FR) may enhance microhabitat heterogeneity (e.g., litter type diversity and soil microtopography variation), increase the number of saprophagous species, and promote their even distribution through niche differentiation. This significantly elevated the Shannon–Wiener index, aligning with the “resource heterogeneity hypothesis” proposed by Stein et al. [54]. In contrast, planting grass (GC) resulted in a simplified necrophagous community structure with dominant species emerging due to limited sources of organic matter (primarily oat residues) and homogenized decomposition environments, which reduced diversity levels. This demonstrates the filtering effect of habitat homogenization on decomposer communities [19]. In summary, ecological restoration measures shape community assembly patterns differently across trophic guilds by regulating key pathways, such as resource availability.

### 4.2. Divergent Roles of Rare and Widespread Species in Community Assembly

This study systematically revealed the differential response patterns of rare and common species to different ecological restoration measures by integrating zeta, dark diversity, and community integrity indices [17] (Figure 4, Figure 5 and Figure 6). Zeta diversity analysis demonstrated that the sharp decline in higher-order zeta values (ζ_2_–ζ_5_) under grazing (CK) and plating grassing (GC) indicates rare species-dominate community succession, reflecting the prevalence of random processes—particularly diffusion limitation, where species dispersal constraints prevent potential colonists from reaching suitable habitats—in highly disturbed environments [34,55]. Grazing disrupts the microhabitats of rare species through trampling activities and resource homogenization, while the monoculture of oats in the fenced pasture planting treatment leads to habitat homogenization [56,57,58]. This makes it difficult for rare species with strict ecological niche requirements to survive sustainably. Conversely, the declining trend in zeta values and the convergence of ratios towards one in the no-till reseeding (FR) and grazing exclusion fencing (FE, phytophagous, and predatory) treatments suggested that common species have become central to community assembly [31]. This is driven by the strengthening of deterministic processes (such as environmental filtering) in a stable resource environment. No-till reseeding (FR) enhances microhabitat heterogeneity, providing common species with diverse ecological niches, while grazing exclusion (FE) preserves host plant stability and ensures resource availability for common species [59].

Furthermore, a comprehensive analysis of dark diversity and community integrity indices confirms the distinct roles of rare and common species in community assembly (Figure 5 and Figure 6). High-probability groups of cryptic diversity (e.g., saprophagous insects in CK and saprophagous and omnivorous insects in GC) suggest that these environments have a large species pool but a low conversion rate [34,60]. This reflects the “source-sink” dynamics of rare species: they can reach these habitats but fail to colonize them successfully due to environmental filtering [61]. Conversely, the combination of low dark diversity probability groups and high CCI values indicates that common species have achieved niche filling and community saturation in suitable habitats [37]. Especially, FR exhibited high CCI values and moderate dark diversity probabilities across all functional groups. This suggests that it supports the persistence of common species and the colonization ability of rare species by creating diverse microhabitats [62]. Therefore, combining zeta diversity and dark diversity in a multidimensional assessment suggests that successful ecological restoration should maintain the stability of common species through resource heterogeneity, while providing rare species with opportunities to colonize through microhabitat diversity. This approach can achieve synergistic recovery of biodiversity across multiple trophic levels.

### 4.3. Dark Diversity as a Novel Metric for Restoration Potential Assessment

Dark diversity analysis provides a new perspective on assessing the potential of ecological restoration measures. This study showed that the high probability of dark diversity under grazing exclusion fencing for herbivorous insects (such as Cicadellidae, Miridae, and Aphididae) suggests that their recovery potential is primarily limited by the availability of host plant resources [63,64,65]. Meanwhile, saprophagous groups (such as Orthocladiinae, *Bradysia lapponica*, and *Scathophaga stercoraria*) exhibit a high probability of high dark diversity under planting grass, which reflects the regulatory role of organic matter quality [66,67,68]. These results validate the importance of dark diversity in quantifying “community saturation” [37]. Notably, the no-till reseeding treatment significantly reduced the dark diversity of omnivorous sensitive species (e.g., *Coenosia verralli*), indicating that habitat improvement can effectively promote niche filling. In contrast, the high dark diversity of saprophagous insects in the grazing control group signifies insufficient recovery. Differential responses to restoration measures by different functional groups suggest that dark diversity can serve as a sensitive indicator of restoration effectiveness [20,69]. The high sensitivity of parasitic wasps (e.g., Pteromalidae) to no-till reseeding reflects the importance of microhabitat heterogeneity, while the weak response of broad-spectrum predators (e.g., Smittia) highlights the buffering effect of ecological niche breadth. These findings imply that regulating key habitat factors, such as host resource availability, microhabitat heterogeneity, and organic matter quality, can differentially shape the potential species pools of different trophic guilds. This provides a scientific basis for developing precise ecological restoration strategies.

### 4.4. Management Implications, Limitations, and Future Prospects

Our study demonstrates that the habitat heterogeneity generated through no-till reseeding effectively promotes multi-trophic insect diversity, offering a viable strategy for enhancing ecosystem resilience in degraded alpine grasslands. To support restoration practice, we recommend prioritizing no-till reseeding in ecologically vulnerable zones, integrating vegetation management with grazing exclusion fencing to prevent over-aggregation of herbivorous insects, and optimizing organic inputs in patch planting to sustain saprophagous insect functions.

We emphasize that while our data reveal clear patterns in insect community responses, the underlying mechanisms require further validation. The correlation between habitat heterogeneity and diversity, though consistent with ecological theory, represents an interpretative conclusion rather than an experimentally verified relationship. Similarly, the proposed resource-based explanations for observed distribution patterns, while plausible, remain to be tested through mechanistic studies. Moreover, several methodological limitations should be considered when interpreting these results. The four-year restoration period, though adequate for detecting initial responses, may not fully represent long-term community trajectories or stable states. In addition, although sweep-netting was standardized across treatments, this method may underrepresent ground-active, nocturnal, or less mobile taxa. The plot-scale design, while enabling controlled comparison, also constrains extrapolation to landscape-level outcomes.

Future research should address these limitations through extended temporal monitoring to evaluate climate restoration interactions, integration of trait-based approaches to elucidate underlying mechanisms, and scaling of dark diversity models to support spatially targeted restoration. Incorporating complementary sampling techniques (pitfall, light trapping, Malaise Trap, etc.) would improve taxonomic representation. Collectively, these advances will help translate theoretical understanding into scalable strategies for sustainable management of alpine grassland ecosystems.

## 5. Conclusions

This study demonstrated that different ecological restoration measures elicit distinct responses from insect communities in degraded alpine grasslands. Our principal finding is that no-till reseeding supports higher multi-trophic diversity across several insect guilds compared to other treatments—a pattern we interpret as being consistent with the habitat heterogeneity generated by this method. In contrast, planting grass initially increased herbivore richness but led to simplified community structure, while grazing maintained higher saprophagous diversity but generally suppressed other functional groups. The integration of zeta and dark diversity analyses provided complementary insights: lower dark diversity under no-till reseeding suggests closer approximation to the potential species pool, whereas higher values under other treatments indicate ongoing recovery deficits.

However, we explicitly distinguish these observed patterns from their mechanistic interpretations. The proposed explanations involving niche partitioning, resource availability, and habitat heterogeneity, while grounded in ecological theory, remain inferential and require direct validation through targeted experimentation. Furthermore, the four-year study period captures initial community responses but is insufficient to evaluate long-term successional trajectories or identify an optimal restoration strategy. Consequently, we recommend prioritizing no-till reseeding with complementary grazing management as a promising approach based on medium-term evidence, rather than a definitive solution. Future research should employ longer temporal monitoring, trait-based analyses, and mechanistic studies to test the hypotheses generated here and establish a more predictive understanding of alpine ecosystem restoration.

## Data Availability

The original contributions presented in this study are included in the article/supplementary material. Further inquiries can be directed to the corresponding authors.

## Supplementary Materials

The following supporting information can be downloaded at: https://www.mdpi.com/article/10.3390/insects16111140/s1.
