# Supplementary material for: Alpine Grassland Ecological Restoration Approaches Shape Insect Trophic Guild Diversity: A Multi-Dimensional Assessment from Alpha to Dark Diversity
Source: Insects. 2025 Nov 7;16(11):1140. doi: 10.3390/insects16111140 (PMC12653117; doi:10.3390/insects16111140)
Supplement: Supplementary file 1 [file insects-16-01140-s001.zip › insects-3897084-supplementary.pdf]

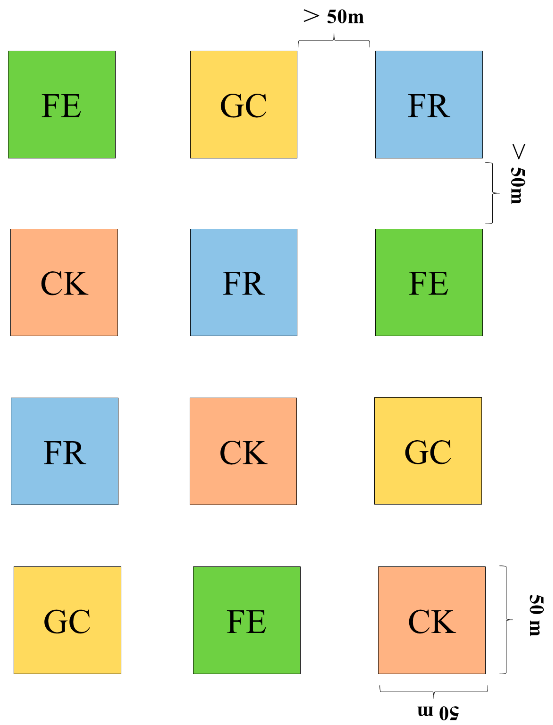

Figure S1. Design of experimental plots in the research area

Note：No-till reseeding (FR), grazing exclusion fencing (FE), planting grass (GC), and grazing management as control (CK)

Table S1. Composition and relative abundance (%) of the main families of insect families in different functional groups under four grassland restoration treatments: planting grass (GC), grazing exclusion fencing (FE), no-till reseeding (FR), and grazing control (CK). Functional groups: phytophagous (Ph), predatory (Pr), saprophagous (Sa), and omnivorous (Om).

**Functional groups**	**Family**	**Treatment**							
		**GC**		**FE**		**FR**		**CK**	
		**No. of individuals**	**Proportion (%)**	**No. of individuals**	**Proportion (%)**	**No. of individuals**	**Proportion (%)**	**No. of individuals**	**Proportion (%)**
Ph	Miridae	40	0.87	3416	**64.70**	1836	**45.00**	42	**7.87**
	Cicadellidae	21	0.46	633	**11.99**	203	**4.98**	31	**5.81**
	Aphididae	98	**2.13**	336	**6.36**	65	**1.59**	17	**3.18**
	Cecidomyiidae	3	0.07	38	0.72	47	1.15	27	**5.06**
	Chrysomelidae	5	0.11	9	0.17	14	0.34	5	0.94
	Chloropidae	0	0.00	22	0.42	10	0.25	4	0.75
Pr	Ceratopogonidae	249	**5.42**	76	1.44	583	**14.29**	79	**14.79**
	Pteromalidae	65	**1.42**	170	**3.22**	182	**4.46**	103	**19.29**
	Ichneumonidae	84	1.83	177	**3.35**	265	**6.50**	50	**9.36**
	Dolichopodidae	271	5.90	12	0.23	53	1.30	10	1.87
	Tachinidae	5	0.11	1	0.02	14	0.34	8	1.50
	Staphylinidae	1	0.02	15	0.28	12	0.29	3	0.56
Sa	Chironomidae	2849	**62.03**	106	**2.01**	226	**5.54**	45	**8.43**
	Scathophagidae	305	**6.64**	30	0.57	126	**3.09**	18	**3.37**
	Sciaridae	26	0.57	66	**1.25**	83	**2.03**	16	**3.00**
	Sepsidae	43	0.94	1	0.02	50	1.23	3	0.56
	Mycetophilidae	37	0.81	15	0.28	30	0.74	6	1.12
	Drosophilidae	30	0.65	3	0.06	2	0.05	4	0.75
Om	Muscidae	363	**7.90**	110	**2.08**	204	**5.00**	50	**9.36**
	Calliphoridae	14	0.30	3	0.06	16	0.39	5	0.94

Table S2. One-way ANOVA results of diversity indices across different functional groups.

**Functional Group**	**Index**	***df***	***F***	**P value**
	Species Richness	11	2.4910	0.0340
	Margalef index	11	1.9116	0.0262
Ph	Shannon-Wiener index	11	1.1558	0.3845
	Pielou index	11	1.1635	0.3820
	Simpson index	11	2.0675	0.1830
	Species Richness	11	2.1102	0.0472
	Margalef index	11	0.1838	0.9044
Pr	Shannon-Wiener index	11	0.2799	0.8385
	Pielou index	11	0.1539	0.9243
	Simpson index	11	0.8309	0.5133
	Species Richness	11	1.2130	0.3659
	Margalef index	11	4.5915	0.0376
Sa	Shannon-Wiener index	11	1.9941	0.0235
	Pielou index	11	2.1940	0.1665
	Simpson index	11	1.4009	0.3117
	Species Richness	11	4.6154	0.0372
	Margalef index	11	0.7075	0.5741
Om	Shannon-Wiener index	11	0.5257	0.6768
	Pielou index	11	0.3865	0.7659
	Simpson index	11	0.1085	0.9528

Note: Functional groups: phytophagous (Ph), predatory (Pr), saprophagous (Sa), and omnivorous (Om). Mean: average value. SE: standard error.

Table S3. Dark diversity probability of insect functional group main species under different grassland restoration measures.

Functional Groups	Species	Treatment (Mean ± SE)			
		CK	FE	FR	GC
Ph	Aphididae	0.59 ± 0.02	0.66 ± 0.02	0.61 ± 0.03	0.65 ± 0.02
	Cecidomyiidae1	0.45 ± 0.03	0.43 ± 0.03	0.34 ± 0.03	0.28 ± 0.04
	Chrysomelidae2	0.37 ± 0.02	0.37 ± 0.02	0.36 ± 0.02	0.35 ± 0.03
	Cicadellidae1	0.89 ± 0.02	0.94 ± 0.00	0.91 ± 0.01	0.84 ± 0.06
	Cicadellidae3	0.38 ± 0.02	0.35 ± 0.03	0.28 ± 0.03	0.21 ± 0.03
	Miridae1	0.62 ± 0.01	0.71 ± 0.02	0.71 ± 0.02	0.63 ± 0.04
	Lygus pratensis	0.28 ± 0.02	0.38 ± 0.02	0.40 ± 0.03	0.55 ± 0.06
Pr	Ceratopogonidae	0.66 ± 0.02	0.65 ± 0.01	0.70 ± 0.01	0.64 ± 0.02
	Hydrophorus altivagus	0.51 ± 0.02	0.61 ± 0.02	0.60 ± 0.02	0.60 ± 0.02
	Lissonota sp.	0.59 ± 0.02	0.60 ± 0.02	0.59 ± 0.01	0.62 ± 0.03
	Stenomacrus sp.	0.81 ± 0.01	0.83 ± 0.01	0.85 ± 0.01	0.80 ± 0.02
	Pteromalidae1	0.46 ± 0.02	0.48 ± 0.01	0.47 ± 0.01	0.48 ± 0.02
	Pteromalidae2	0.82 ± 0.01	0.85 ± 0.01	0.84 ± 0.00	0.80 ± 0.02
Sa	*Orthocladiinae sp.*	0.91 ± 0.01	0.88 ± 0.01	0.91 ± 0.01	0.90 ± 0.01
	Rymosia fasciata	0.53 ± 0.06	0.63 ± 0.02	0.68 ± 0.04	0.71 ± 0.03
	Scathophaga stercoraria	0.61 ± 0.04	0.64 ± 0.03	0.68 ± 0.04	0.67 ± 0.07
	Bradysia lapponica	0.78 ± 0.01	0.76 ± 0.01	0.77 ± 0.02	0.74 ± 0.01
	Sepsis neglecta	0.45 ± 0.04	0.48 ± 0.02	0.55 ± 0.04	0.58 ± 0.02
Om	Calliphoridae2	0.37 ± 0.03	0.34 ± 0.01	0.36 ± 0.02	0.41 ± 0.03
	Muscidae1	0.27 ± 0.03	0.28 ± 0.01	0.30 ± 0.01	0.33 ± 0.03
	Muscidae2	0.51 ± 0.12	0.65 ± 0.10	0.80 ± 0.00	0.80 ± 0.01
	Coenosia verralli	0.71 ± 0.07	0.79 ± 0.03	0.84 ± 0.01	0.84 ± 0.02

Note: Restoration treatments: planting grass (GC), grazing exclusion fencing (FE), no-till reseeding (FR), and grazing control (CK). Functional groups: phytophagous (Ph), predatory (Pr), saprophagous (Sa), and omnivorous (Om). Mean: average value. SE: standard error.
